# Therapeutic update in the treatment of disruptive disorder with emotional dysregulation in children and adolescents : review of the literature

**DOI:** 10.1192/j.eurpsy.2023.1512

**Published:** 2023-07-19

**Authors:** H. Belhadga, Z. Elmaataoui, H. Kisra

**Affiliations:** Hôpital psychiatrique universitaire arrazi de Sale, Hôpital psychiatrique universitaire arrazi de Sale, Sale, Morocco

## Abstract

**Introduction:**

Disruptive Mood Dysregulation Disorder (DMDD) is a new disorder that has been added to the category of mood disorders in the fifth Diagnostic and Statistical Manual of Mental Disorders to distinguish chronic non-periodic irritability from the periodic irritability of bipolar disorder. The main characteristic of DMDD is chronic and severe irritability. Because it is a new diagnostic entity, little research has been done on it and the literature on the subject is still expanding.

**Objectives:**

The purpose of this review article is to gather information on new therapies for the treatment of this disorder in children and adolescents.

**Methods:**

The studies related to the treatment of DMDD were collected and analyzed. This study retrieved related articles from PubMed, SpringerLink, ScienceDirect, NCBI, The American Journal of Psychiatry, and EBSCO. Use keywords “disruptive” AND “mood” AND “dysregulation” AND “disorder” OR “Treatment” AND “DMDD” OR “Drug” AND “mood” AND “disorder” OR “Treatment” AND “SMD” OR “Treatment” AND “BP” OR “Treatment” AND “ADHD” OR “Antidepressant” OR “Mental”AND “Stabilizer” OR “temper” AND “outburst” OR “aggressive” AND "antipsychotics.

**Results:**

To date, no medication has been approved by the FDA to treat EDD. Because there are no treatment standards, drug therapy focuses on the primary symptoms of EDD, such as severe chronic irritability, temper tantrums, and comorbidities, such as ADHD. Currently, medications used by clinicians to treat patients with EDD include antidepressants (fluoxetine, sertraline, citalopram), stimulants (methylphenidate), anxiolytics mood stabilizers (sodium valproate) and antipsychotics (haloperidol, risperidone, aripiprazole in combination with methylphenidate in ADHD-EDD comorbidity), atomoxetine, guanfacine, and amantadine.

To date, no medication has been approved by the FDA to treat EDD. Because there are no treatment standards, drug therapy focuses on the primary symptoms of EDD, such as severe chronic irritability, temper tantrums, and comorbidities, such as ADHD. Currently, medications used by clinicians to treat patients with EDD include antidepressants (fluoxetine, sertraline, citalopram), stimulants (methylphenidate), anxiolytics mood stabilizers (sodium valproate) and antipsychotics (haloperidol, risperidone, aripiprazole in combination with methylphenidate in ADHD-EDD comorbidity), atomoxetine, guanfacine, and amantadine.

**Conclusions:**

As a new diagnosis, treatment guidelines for DMDD are still unclear. Preliminary results from this study suggest that clinicians tend to prescribe a variety of psychotropic medications. This heterogeneity in treatment choices may reflect the fact that these patients are on a bridge between disruptive behavior disorders (including ADHD) and mood disorders. The relative merits or demerits of these treatment choices should be evaluated in further studies.

**Disclosure of Interest:**

None Declared

